# A comparison of BeadChip and WGS genotyping outputs using partial validation by sanger sequencing

**DOI:** 10.1186/s12864-020-06919-x

**Published:** 2020-09-10

**Authors:** Kirill A. Danilov, Dimitri A. Nikogosov, Sergey V. Musienko, Ancha V. Baranova

**Affiliations:** 1 Atlas Biomed Group Limited, Tintagel House, 92 Albert Embankment, Lambeth, London, SE1 7TY UK; 2 Skolkovo Institute of Science and Technology, Bolshoy Boulevard 30, bld. 1, 121205 Moscow, Russia; 3 School of Systems Biology, George Mason University, 10900 University Blvd, Fairfax, VA 22030 USA; 4 Research Center for Medical Genetics, Moskvorechye St., 1, 115478 Moscow, Russia

**Keywords:** WGS, WES, Whole genome sequencing, Microarray genotyping, Genotype concordance, Sanger sequencing

## Abstract

**Background:**

Head-to-head comparison of BeadChip and WGS/WES genotyping techniques for their precision is far from straightforward. A tool for validation of high-throughput genotyping calls such as Sanger sequencing is neither scalable nor practical for large-scale DNA processing. Here we report a cross-validation analysis of genotyping calls obtained via Illumina GSA BeadChip and WGS (Illumina HiSeq X Ten) techniques.

**Results:**

When compared to each other, the average precision and accuracy of BeadChip and WGS genotyping techniques exceeded 0.991 and 0.997, respectively. The average fraction of discordant variants for both platforms was found to be 0.639%. A sliding window approach was utilized to explore genomic regions not exceeding 500 bp encompassing a maximal amount of discordant variants for further validation by Sanger sequencing. Notably, 12 variants out of 26 located within eight identified regions were consistently discordant in related calls made by WGS and BeadChip. When Sanger sequenced, a total of 16 of these genotypes were successfully resolved, indicating that a precision of WGS and BeadChip genotyping for this genotype subset was at 0.81 and 0.5, respectively, with accuracy values of 0.87 and 0.61.

**Conclusions:**

We conclude that WGS genotype calling exhibits higher overall precision within the selected variety of discordantly genotyped variants, though the amount of validated variants remained insufficient.

## Background

Both Whole Genome (WGS) and Whole Exome sequencing (WES) are now used in multiple avenues of clinical and scientific inquiry. Despite increased availability of these techniques and rapid decline of associated costs, their context-dependent per-base performance remained in question. The performance characteristics of WGS/WES include accuracy (the extent of agreement between the reference and the assay-derived nucleic sequence), precision which is broadly defined as repeatability for within-run precision and reproducibility for between-run precision as well as analytical sensitivity, specificity and a reportable range of the reference genome coverage [1]. While the repeatability issues were extensively presented in detail previously [2], the absence of scalable non-NGS (next-generation sequencing) techniques for genotype calling limits evaluations of accuracy.

BeadChip genotyping is an efficient and scalable way of genotype resolution, with two inherent limitations: a necessity to decompose binary alleles and confinement to a predefined list of genotyped variants. These two limitations, however, do not prevent its usefulness for a variety of clinical and non-clinical applications [3]. Predefined nature of the variants to be tested makes BeadChip genotyping amenable to validation by either PCR (polymerase chain reaction), or Sanger sequencing which has been recently shown to have limited utility and erroneous behaviour in the validation of NGS variants [4]. A comparison of genotyping calls made by BeadChip and WGS/WES techniques may provide an insight into the possible nature of the discordant calls observed during genotyping quality control stage. Here we attempted to identify analytical issues leading to the discordance in genotyping calls made independently by WGS and BeadChip techniques.

## Results

### Sequencing statistics

Table 1 lists the sequencing statistics for the three sequenced samples. FastQC reports are available as Additional files 1, 2, 3, 4, 5 and 6 for sample_001, sample_002, sample_003, respectively. Percentage of reads falling into a category with averaged Phred scaled sequencing quality above 30 is shown by %Q. The absence of unpaired reads, repeatability in terms of GC content and more than 90% of bases exceeding sequencing quality of 30 was used as a mark of confidence in data quality.

**Table 1:** Raw sequencing data statistics summary

Sample	Read orientation	Mean read quality (Phred score)	Number of reads	% GC	% Q > 30	Number of bases (1e6)	Mean read length (bp)
001	R1	39.31	399036358	41.07	95.56	59855.45	150.0
	R2	36.76	399036358	41.21	86.17	59855.45	150.1
002	R1	39.28	375900168	41.06	95.47	56385.03	150.2
	R2	36.8	375900168	41.22	86.32	56385.03	150.3
003	R1	39.32	385826012	40.95	95.59	57873.9	150.4
	R2	36.49	385826012	41.08	85.15	57873.9	150.5

The table describes the raw sequencing data quality metrics for all sequenced samples; *GC* Guanine-cytosine sequence content

### Mapping statistics

**Fig. 1:**
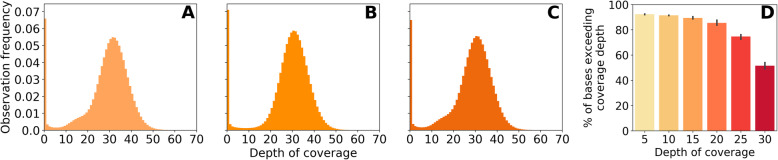
Whole genome depth of coverage distributions. Metrics for sample_001 (**a**), sample_002 (**b**), sample_003 (**c**) and breadth of coverage for the specified depth thresholds (**d**) averaged for all three samples are shown with 95% confidence intervals, *n* = 3

All the data produced by WGS were analyzed for their depth (DOC) and breadth (BOC) of coverage using GATK 3.8 DepthOfCoverage tool [5]. The mean filtered coverage for all three samples exceeded 27x (Fig. 1a–c, Table 2), which complied with recommendations [6]. Repeatability in BOC values for each base quality interval (Fig. 1b, Table 2) and other sequencing metrics (Table 1) proved the quality of the sequencing.

**Table 2:** WGS coverage statistics for the filtered mapped data

Sample	Total filtered bases	Mean DOC	% bases above 10	% bases above 20	% bases above 30
001	87347116777	28.70	91.6	84.8	54.1
002	86399503195	28.39	91.3	87.6	51.3
003	84678940424	27.82	91.5	83.8	49.1

### Concordance metrics

**Fig. 2:**
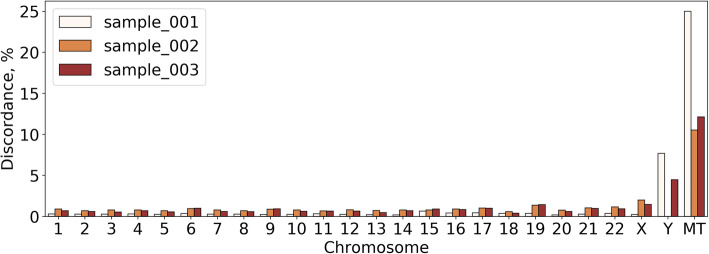
Fractions of discordant results for three samples. Percentage of discordant results per each chromosome is shown where applicable

Percentages of discordant calls per chromosome for each sample are shown in Fig. 2. The average fraction of discordant results among all comparable genotyping results (intersection of BeadChip and WGS obtained genotyping data) was at 0.3317% for sample_001 (male), 0.8448% for sample_002 (female) and 0.7392% for sample_003 (male) which falls within the range reported previously [2]. Both the sequencing quality and mapping quality parameters were reproduced for all three sequenced samples with an average genotype concordance estimate being higher than 99%.

### Mapping of analyzed variants

**Fig. 3:**
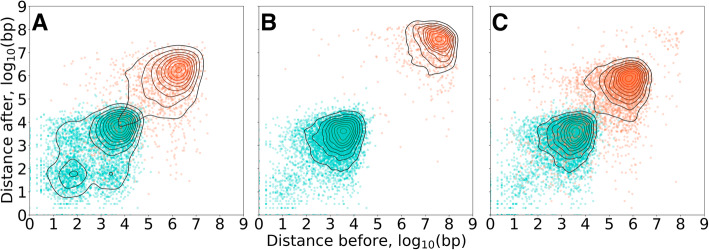
Distance maps for the analyzed samples. **a** — sample_001, **b** — sample_002, **c** — sample_003, concordant and discordant variants are marked in green and orange, respectively

For each pair of BeadChip-genotyped neighboring variants, distance intervals were extracted within both concordant and discordant group to generate pairs of distance values before and after each variant, followed by their visualization. The resulting maps with a Gaussian kernel density estimation are shown in Fig. 3. The observed approximate evenness of distribution for both concordant and discordant variants supported by the visible clusters allocation along the bisectrix of the axes shows that both concordant and discordant variants are evenly distributed across the chromosome length, with no congregation across the genome. The only observed difference in cluster allocation arises from the frequency of observation for the variants from each group. In other words, less frequent discordant group corresponds to lower mapping density and, consequently, to more considerable distances between every two neighboring variants.

Overall randomness of the locations of discordant genotypes across the genome, measured as cluster evenness and cluster center distance from the bisectrix, was high. However, because locations of discordant variants were limited to variants present on the corresponding BeadChip, the comparison with WGS was necessarily limited to the locations of BeadChip genome variants only. For this reason, patterns in discordant genotypes location throughout the genome were only detectable at locations thoroughly covered by variants presented at BeadChip, which generally are outside any complex genomic regions. Because of that, it was unlikely to detect any unevenness in the WGS-derived discordant genotype calls distribution.

### Concordance analysis

**Fig. 4:**
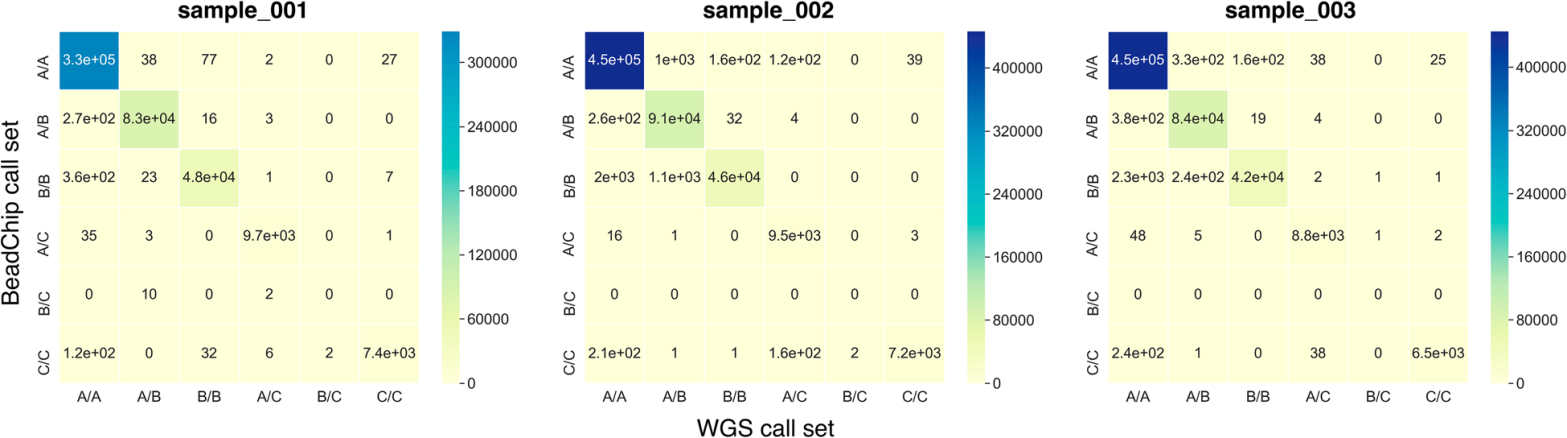
Confusion matrices calculated for the call sets obtained by WGS and BeadChip. WGS was defined as “true” call set, BeadChip — “test” call set, data is shown for sample_001 (male, chromosomes MT, X, Y were excluded from analysis), sample_002 (female, chromosome MT was excluded from analysis) and sample_003 (male, chromosomes MT, X, Y were excluded from analysis)

Calculated confusion matrices for all three analyzed DNA samples are shown in Fig. 4, with metrics values presented in Table 3. These values show high overall concordance in genotyping between both WGS and BeadChip techniques. Detected discordant results may have arisen from ambiguous BeadChip genotyping call within multiallelic sites. Nevertheless, all calculated quality metrics values surpassed the recommended thresholds [6].

**Table 3:** Quality metrics of genotyping comparison between BeadChip and WGS call sets

Sample	Genotype concordance	Non-reference genotype concordance	Non-reference genotype sensitivity	Sensitivity	Precision	Specificity	Accuracy
001	0.99783	0.99616	0.99687	0.99783	0.99783	0.99925	0.99928
002	0.99156	0.97973	0.98773	0.99156	0.99156	0.99696	0.99718
003	0.99253	0.985	0.98402	0.99253	0.99253	0.99742	0.9975

### Genotyping quality metrics distributions

**Fig. 5:**
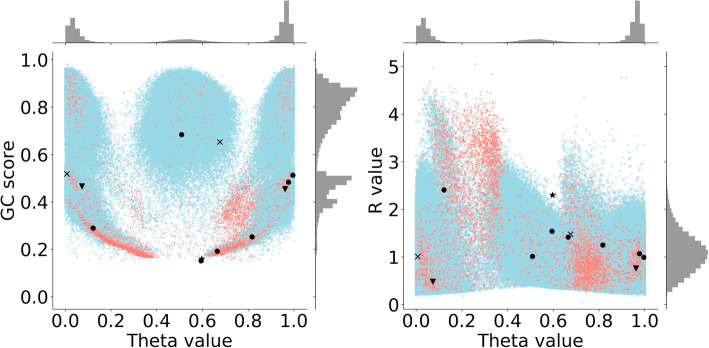
BeadChip genotyping quality metrics with highlighted Sanger-validated variants. Theta, R, GC Score values for sample_002 are shown; histograms show the corresponding distributions of plotted metrics in a 1-dimensional space; concordant and discordant variants are marked in blue and orange, respectively; genotypes which are not consistent with Sanger sequencing in both WGS and BeadChip results are marked with a star, matches between Sanger and BeadChip are marked with triangles, matches between Sanger and WGS are marked with circles, variants which were not successfully genotyped by Sanger are marked with crosses

For all three samples, the distributions of the WGS genotyping metrics were analyzed and compared within the groups formed based on the concordance, variation class and genotype zygosity criteria (data not shown). While differences in mean values were statistically significant (Welch’s t-test, 0.05 *p*-value threshold) in several groups, distributions themselves exhibited low intergroup divergence. Moreover, for all group comparisons, observed differences were quite small, and their patterns were not uniform, with several groups being insufficiently large, potentially obscuring inter-group dissimilarity. Thus, discordance in genotyping could not be explained by the variance in these quality metrics. When quality metrics were analyzed for the BeadChip calls, some differences in parameters distributions were found. The distinction was remarkable only for the SNV group, as it included the significant amount of all genotyping results and thus could more precisely represent any actual deviations. Inspection of R, Theta and GC scores for both concordant and discordant variants revealed a pattern of discordant variants located close to the borders of the variant clusters (Fig. 5). Importantly, similar clusters are used in the Illumina GenomeStudio software to assign genotypes to variants (AA, AB and BB), and, therefore, any genotype lying far from the cluster centre may be mistakenly assigned an irrelevant designation. This explanation for the observed discordance pattern was, however, relevant only to sample_002 and sample_003. In sample_001, distribution of discordant variants within the clusters was more or less even. Therefore, we conclude that the observed phenomenon requires further examination. Potential finding of the clustering pattern for discordant calls may then be exploited for quality control.

### Sanger sequencing

The sliding window approach performed on sample_002 resulted in the mapping of 6 regions containing from 1 to 3 variants successfully genotyped on both WGS and BeadChip platforms and exhibiting discordant genotypes between the used platforms. Locations of these regions and encompassed variants are collated in Tables 4 and 5. A total of 12 discordant variants were selected for validation by Sanger sequencing. These variants were accompanied by 14 concordant variants located closely to discordant variants. These 12 discordant variants included 6 SNVs and 6 INDELs, of which 4 SNVs were of AA vs BB type discordancy, 2 — AA vs AB type, 3 INDELs of II vs DD type and the remaining 3 INDELs of DD vs DI (II vs DI) type. The selection was performed based on the following criteria:

1. Selected SNPs were successfully genotyped using both WGS and BeadChip platforms.
2. Selected SNPs are located close to each other within 500 bp window length (reasonable limit of one Sanger read).
3. Specific primers can be selected for these SNPs-containing region (by Primer-BLAST [7]).

**Table 4:** The chosen regions for validation by Sanger sequencing for sample_002

Chromosome	Start	End	INDELs	SNVs	Total	Genotypes		Vartype
						BeadChip	WGS	
1	109711850	109712150	0	3	3	TG	GG	3 SNVs
						TC	TT	
						CG	GG	
1	109759300	109759400	1	0	1	DD	II	1 INDEL
2	85663600	85663700	1	0	1	DI	II	1 INDEL
2	233671750	233671850	1	0	1	II	DI	1 INDEL
6	160246180	160246690	0	3	3	GG	AA	3 SNVs
						TT	CC	
						GG	AA	
10	101010500	101010600	1	0	1	DD	II	1 INDEL
13	49792500	49792600	1	0	1	DD	DI	1 INDEL
22	37973800	37974170	1	0	1	DD	II	1 INDEL

**Table 5:** The designed primers for amplification and sequencing of the chosen regions

Chromosome	Orientation	Name	Sequence 5′3’	Length	Tm	GC	Start	End	Amplifying length	N mismatches	Mismatch type
1	F	chr1_reg1_F	AAGCCCTCGGAGTAGCTTTC	20	59.46	55	109711755	109711774	397	3	3 SNV
1	R	chr1_reg1_R	GGCTGGAATCAATAAGCCCC	20	58.67	55	109712151	109712132	397	3	3 SNV
1	F	chr1_reg2_F	TGATGGACAGGATGGAGTTGTAG	23	59.55	47.83	109759300	109759322	85	1	1 INDEL
1	R	chr1_reg2_R	GAGCTGGACTCTTACCGCCTA	21	61.02	57.14	109759384	109759364	85	1	1 INDEL
2	F	chr2_reg1_F	GTGGTCACGGACATGCAGA	19	60.01	57.89	85663428	85663446	344	1	1 INDEL
2	R	chr2_reg1_R	CTGAGCGCTACTCCGTCATC	20	60.32	60.00	85663771	85663752	344	1	1 INDEL
2	F	chr2_reg2_F	CTCTGGACAGAGAGTATTTGGTTG	24	58.83	45.83	233671510	233671533	370	1	1 INDEL
2	R	chr2_reg2_R	AGGTGGGAGAAATACCAGCAC	21	59.72	52.38	233671879	233671859	370	1	1 INDEL
6	F	chr6_F	TTTAAGAAGGCAGGGGATTGCT	22	59.96	45.45	160246431	160246452	490	3	3 SNV
6	R	chr6_R	GCAACTTAAGCCTTCACCAGT	21	58.77	47.62	160246920	160246900	490	3	3 SNV
10	F	chr10_F	CCTGGAGACTTGCCTTGACC	20	60.32	60	101010511	101010530	233	1	1 INDEL
10	R	chr10_R	CCTCTACAAGACGTGCCAGT	20	59.40	55	101010743	101010724	233	1	1 INDEL
13	F	chr13_F	AAGCTCTTGATGCGGTGGTT	20	60.25	50	49792463	49792482	330	1	1 INDEL
13	R	chr13_R	CACGTATAGCCCGGCGAA	18	59.59	61.11	49792792	49792775	330	1	1 INDEL
22	F	chr22_F	CGAGGGCCCCATATAGGAGA	20	59.96	60	37973603	37973622	408	1	1 INDEL
22	R	chr22_R	GAGTTGGACCAGTACCTGCC	20	60.04	60	37974010	37973991	408	1	1 INDEL

The list of designed primers with respective amplification parameters can be found in Table 5. The gDNA of sample_002 was used for amplifying regions of interest (ROIs) in Table 4. All ROIs, except those located on chromosomes 10 and 13, were successfully amplified and Sanger sequenced. The comparison of genotypes obtained via microarray genotyping, whole genome sequencing and Sanger sequencing of the amplified ROIs is shown in Table 6. Sanger-derived genotypes containing only one letter were the ones obtained from only one read. Plus signs denote Sanger genotypes concordant with the WGS-derived genotype, while double plus signs denote concordance of all three genotyping methods. Hashtag represents concordance with the BeadChip-derived genotype, and asterisk — an absence of concordance with any of the tested methods. Sixteen Sanger-resolved diploid genotypes (forward and reverse Sanger chromatograms covered the variant location) out of the 26 listed in Table 6 were used for confusion matrices calculation using Sanger-derived calls as a “truth” call set, which resulted in WGS and BeadChip precision of 0.81 and 0.5, respectively, and the accuracy values of 0.87 and 0.61. Results of Sanger validation may possibly be explained by low complexity or repetitive genomic context which surrounds some of the validated variants, hindering accuracy and precision of either genotyping or read alignment. All the listed variants which were discordant between BeadChip and WGS were plotted (Fig. 5) to reveal possible clustering of discordant variants based on the initial BeadChip genotyping metrics. No clustering of the validated variants was observed. Thus, current analysis did not allow to make any definite conclusions about the clustering of discordant and concordant variants based on their genotyping quality metrics.

**Table 6:** Sanger validation results for sample_002

rsID	Chromosome	Position (GRCh38)	BeadChip genotype	WGS genotype	Sanger genotype
rs12731384^d^	1	109711898	TG	GG	TT
rs1633365^c^	1	109711919	TC	TT	TC
rs58877308^a^	1	109712113	CG	GG	GG
rs138687644	1	109759369	GG	GG	G–
rs202018423^b^	1	109759339	CC	CC	CC
rs757293027	1	109759384	CC	CC	C–
rs760279355^b^	1	109759346	CC	CC	CC
rs767809812	1	109759359	DD	II	I–
rs79394341	1	109759361	GG	GG	G–
rs786205634^a^	2	85663647	DI	II	II
rs3832043^d^	2	233671807	II	DI	DD
rs144484152^a^	6	160246683	GG	AA	AA
rs149262397^b^	6	160246574	CC	CC	CC
rs316022^a^	6	160246568	GG	AA	AA
rs529525717^b^	6	160246473	GG	GG	GG
rs537568133^a^	6	160246590	TT	CC	CC
rs549969754^b^	6	160246607	CC	CC	CC
rs555024471	6	160246297	GG	GG	G–
rs563560445^b^	6	160246467	CC	CC	CC
rs563829592	6	160246443	GG	GG	G–
rs577352795	6	160246460	TT	TT	T–
rs757852385^b^	6	160246688	AA	AA	AA
rs759566284	6	160246458	GG	GG	G–
rs200896335	10	101010536	DD	II	NA
rs753420953	13	49792587	DD	DI	NA
rs397515367^a^	22	37973817	DD	II	II

^a^concordance between Sanger and WGS genotypes

^b^concordance between all three genotyping methods

^c^concordance between Sanger and BeadChip genotypes

^d^absence of concordance between any of the methods

## Discussion

Although genotype concordance analysis experiments using different sequencing and genotyping platforms have been performed previously [2, 8], no reports on attempts to explain the observed differences were made. Here we tried to scrutinize the underlying genotyping process proxy such as genotyping quality metrics to find a possible explanation for the discordance pattern. Unfortunately, the genotyping discordance of the observed levels (less than 1%) is usually an underestimation, and does not motivate investigations into the nature of the discordant genotype calling. However, we show that discordant genotypes tend to form clusters in a 2-dimensional space, where the most vivid dimensions are the genotyping quality and technical metrics obtained from the BeadChip genotyping pipeline. Therefore, speculation on possibly lower precision of the BeadChip genotyping platform, as compared to WGS-based pipelines, might find a new conceivable basis upon further investigation. Genotype assignment in the array pipelines is based on marker clustering in a 2-dimensional space (clusters A/A, A/B, B/B), which might happen to be erroneous due to poor cluster separation. This clusterization problem may possibly be solved by incorporating additional dimensions into the analyzed genotyping metrics space, which can be exploited for enhancement of the BeadChip genotyping pipeline upon further investigation and vast Sanger validation of the variants.

## Conclusions

Here we show the presence of some parametric differences in quality metrics of genotyping performed by WGS and BeadChip. This phenomenon warrants comprehensive investigation by combining genotyping metrics produced by WGS and BeadChip pipelines and extracting patterns in the observed discordance. In clusters, Sanger validation should be performed for genotype resolution.

## Methods

### Materials

Three human genomic DNA samples, two males and one female, were selected for this comparison. After collection by Oragene DNA saliva-based collection device (DNA Genotek, Canada), genomic DNA was extracted as per the manufacturer protocol and stored frozen in Tris-EDTA buffer at − 20 °C until genotyping was performed.

### BeadChip genotyping

Infinium iSelect 24 × 1 HTS Custom Beadchip Kit (GSAsharedCUSTOM_20018389_A2) genotyping was performed using 50 ng of genomic DNA. Microarray fluorescence was scanned using the Illumina iScan system, and the genotype calling was executed using the Illumina GenomeStudio Genotyping Module software (Illumina, USA). No imputation was implemented, and only variants successfully genotyped on the specified array were used in the analysis.

### Genome sequencing and variant calling

Whole genome sequencing was performed by MedGenome (CA, USA) using the HiSeq X Ten platform (Illumina, USA) utilizing a 150 base-pair (bp) paired-end protocol. The raw FASTQ files were evaluated using FastQC software (Babraham Institute, UK). A proprietary WGS data processing pipeline was designed to process NGS data in automated mode from raw FASTQ files to finalized genotyping data. The pipeline implemented adapter trimming using Trimmomatic [9], BWA MEM [10] alignment to the GRCh38 build of the human genome, duplicate reads marking by Picard (Broad Institute, USA), filtering of the resulting SAM/BAM files via SAMtools [11, 12]. It also utilized GATK tools (Broad Institute, USA) to perform base quality score recalibration (BQSR), genotype calling and variant quality score recalibration (VQSR). GATK VariantAnnotator and BCFtools were employed to perform variant annotation. The pipeline implemented several hard filtering procedures resulting in a final VCF output with all variant and non-variant sites which passed quality control of the filtering step. The pipeline was designed in compliance with GATK Best Practices recommendations for NGS data processing. Single nucleotide variants (SNVs) and indels were called using GATK HaplotypeCaller with subsequent VQSR using a threshold of 99.9, HapMap 3.3, 1000G Omni 2.5, 1000G Phase 1 High Confidence and dbSNP build 151 training sets for SNV mode and Mills and 1000G Gold Standard Indels with dbSNP build 151 training sets for INDEL mode.

### Quality metrics and validation statistics

**Fig. 6:**
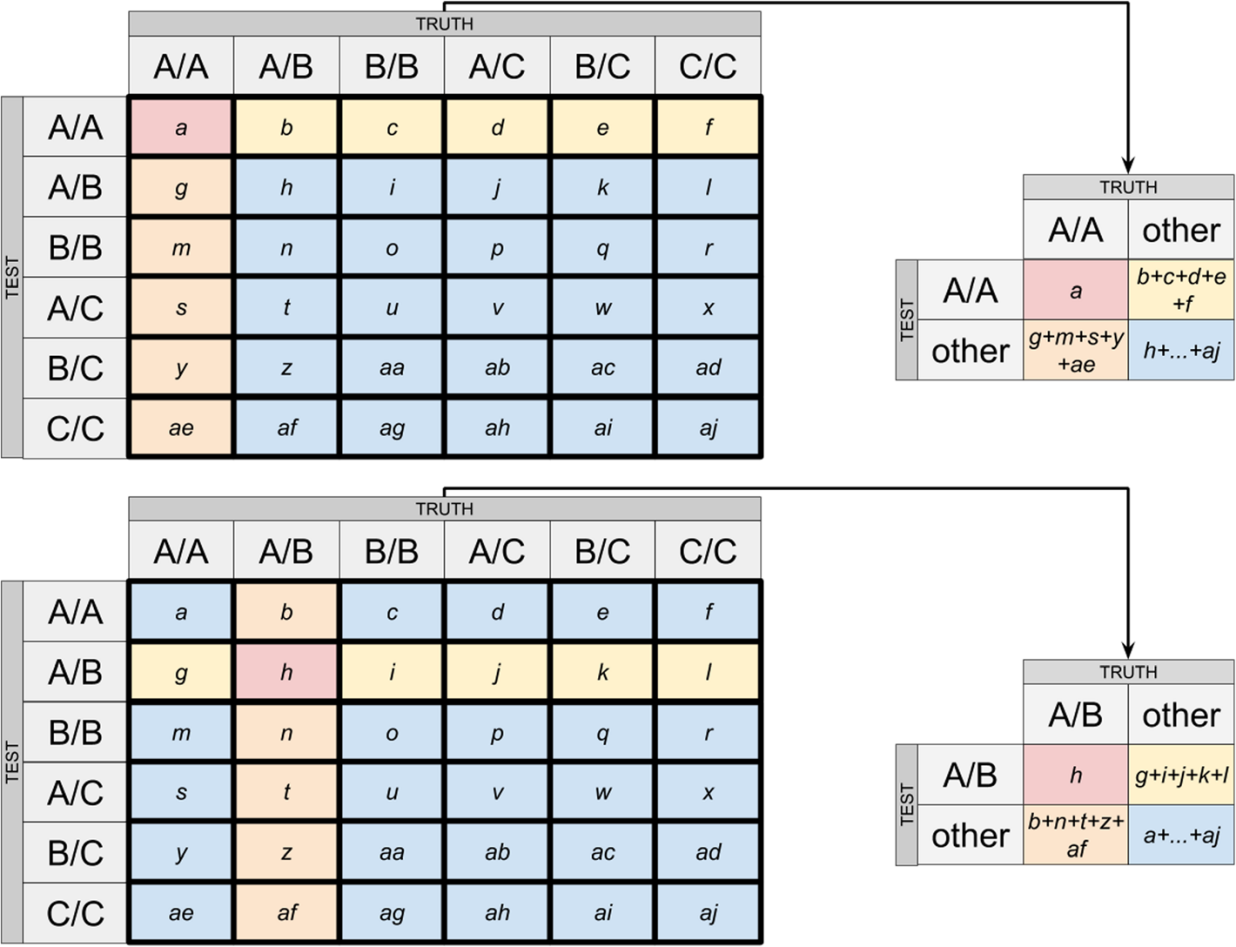
Example calculation of confusion matrices. The shown dimensionality reduction is used for accuracy and other metrics calculation; a, b, c, d, …, ai, aj — sample counts of each class; A/A, A/B, B/B, A/C, B/C, C/C — diploid genotypes observed in data; A — reference allele, B and C — alternative alleles. TRUTH — a call set produced by an orthogonal method (comparator), TEST — a call set produced by a test method

**Fig. 7:**
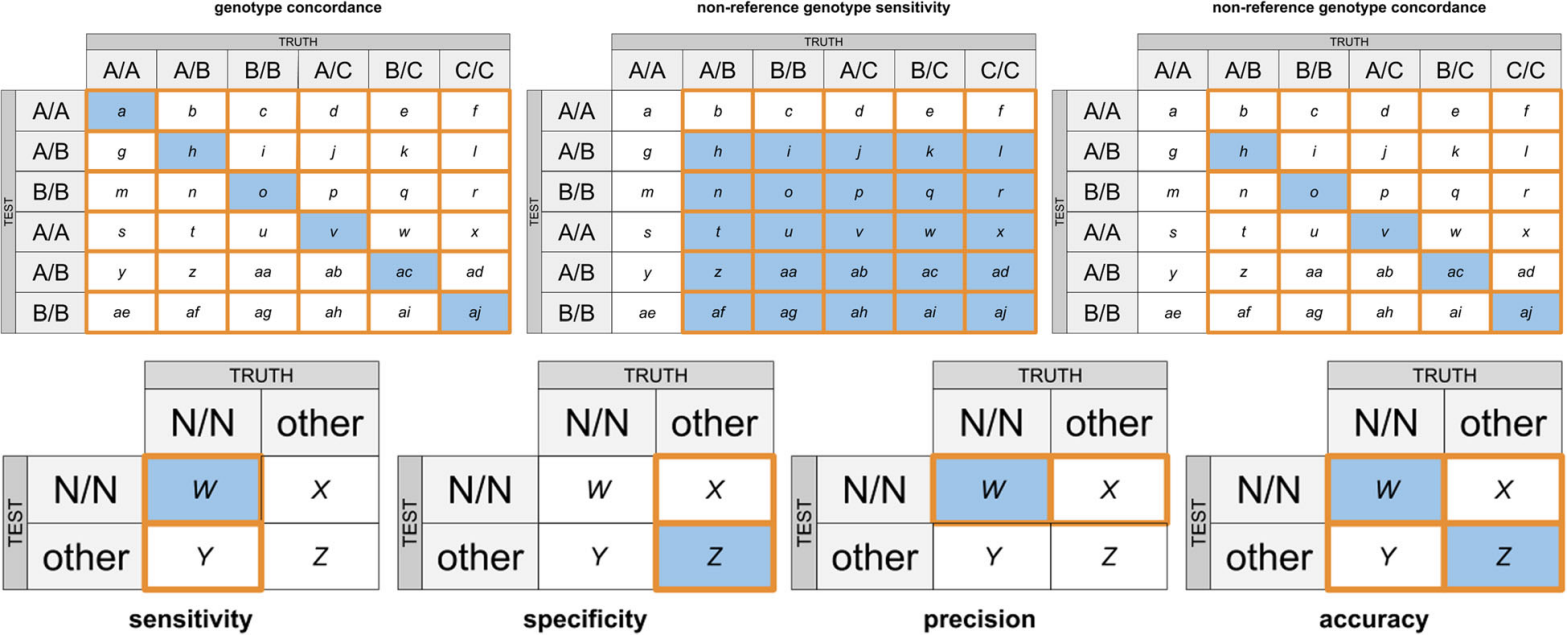
Quality metrics calculation for the initial and the “reduced confusion” matrices. Each metric is calculated as a ratio of blue elements to orange-outlined elements; A/A, A/B, B/B, A/C, B/C, C/C — diploid genotypes observed in data; A — reference allele, B and C — alternative alleles; N/N — any diploid genotype category (A/A, A/B, B/B, A/C, B/C, C/C). TRUTH — a call set produced by an orthogonal method (comparator), TEST — a call set produced by a test method

Concordance analysis implied calculation of specificity, sensitivity, precision, accuracy, genotype concordance, non-reference genotype concordance and non-reference sensitivity of genotyping between BeadChip and WGS. The analysis implemented confusion matrices calculation (Fig. 6). As there is no baseline “truth” when a WGS call set is compared with a BeadChip one, no method can be described as “comparator”. Because of this, each call set within each pair was treated as alternating “truth” or “test” call set, followed by the averaging of obtained statistics. Filling those matrices with observed numbers of class counts was followed by dimensionality reduction, which implied leaving one class in both call sets intact and combining the counts in all other classes (Fig. 6). The above-specified quality metrics for the initial and reduced matrices were calculated, as shown in Fig. 7 [2] with amendments. As each non-reduced matrix produced 6 submatrices, the calculated sensitivity and precision value for each submatrix was weighted by the fraction of analyzed elements in a non-reduced matrix (i.e., each calculated metric was then multiplied by the ratio of orange-outlined elements to all elements in the reduced matrix in Fig. 7). Such an approach was only necessary for precision and sensitivity metrics due to the fact that these metrics were calculated on non-overlapping subsets of the initial matrix and their values depended on the size of those subsets, given uneven marker distribution in the initial matrix. Other used metrics (accuracy, specificity) were calculated as mean values between 6 produced metric values (excluding NANs, which originated from calculations involving division by zero).

All concordant and discordant genotyped variants were analyzed for genotyping quality metrics provided in the VCF files and GenomeStudio report files for WGS and BeadChip genotyping, respectively. The following quality metrics obtained via WGS genotyping pipeline were analyzed:

1. DP variant read depth at a particular position for a particular sample.
2. QUAL Phred-scaled quality score for the assertion made in ALT. i.e., *−10log*_*10*_*(P*_*call in ALT is wrong*_*)*, if ALT is “.” (no variant) then this is *–10log*_*10*_*(P*_*variant*_*)*, and if ALT is not “.” this is *–10log*_*10*_*(P*_*no variant*_*).*
3. RGQ unconditional reference genotype confidence, encoded as a Phred quality *–10log*_*10*_*(P*_*genotype call is wrong*_*).*
4. GQ conditional genotype quality, encoded as a Phred quality *–10log*_*10*_*(P*_*genotype call is wrong*_*),* conditioned on the site is variant.

The following metrics were extracted from corresponding GenomeStudio reports:

1. GC score GenCall score, a quality metric that indicates the reliability of each genotype call. The GenCall Score is a value between 0 and 1 assigned to every called genotype. Genotypes with lower GenCall scores are located further from the centre of a cluster and have lower reliability.
2. GT score GenTrain score, a quality metric that indicates how well the samples clustered for a locus.
3. Cluster Sep cluster separation score.
4. Theta — the normalized Theta-value of the SNP for the sample.
5. R — the normalized R-value of the SNP for the sample.
6. X — the normalized intensity of the A allele.
7. Y — the normalized intensity of the B allele.
8. B Allele Freq B allele theta value of the SNP for the sample, relative to the cluster positions. This value is normalized so that it is zero if theta is less than or equal to the AA cluster’s theta mean, 0.5 if it is equal to the AB cluster’s theta mean, or 1 if it is equal to or greater than the BB cluster’s theta mean. B Allele Freq is linearly interpolated between 0 and 1.

### Variant selection for sanger sequencing

An intersection of WGS- and array-genotyped markers which exhibited discordant calls between the two platforms was used for selection. A sliding window approach was utilized to find regions spanning no more than 500 bp (Sanger sequencing reasonable read length limit) and encompassing as many discordant variants from the selected set as possible. All primers for PCR amplification were designed using NCBI Primer-BLAST suite [7] with default parameters (except the melting temperature limits of 58–62 °C) and the human genome reference sequence for BLAST. PCR product lengths and primer lengths were manually optimized to find the most suitable unique match. Primer synthesis, amplification and Sanger sequencing was performed by Evrogen (Russia, Moscow). Sanger chromatograms were visualized and analyzed using 4Peaks software (Nucleobytes, The Netherlands) and CodonCode Aligner (CodonCode Corp., USA) with default trimming and quality filtering parameters.

## Supplementary information

**Additional file 1.** FastQC report for forward reads.

**Additional file 2.** FastQC report for reverse reads.

**Additional file 3.** FastQC report for forward reads.

**Additional file 4.** FastQC report for reverse reads.

**Additional file 5.** FastQC report for forward reads.

**Additional file 6.** FastQC report for reverse reads.

## Data Availability

The datasets generated and/or analysed during the current study are not publicly available due Atlas Biomed personal data privacy policy but are available from the corresponding author on reasonable request.

## Abbreviations

WGS: Whole genome sequencing
WES: Whole exome sequencing
NGS: Next-generation sequencing
PCR: Polymerase chain reaction
DOC: Depth of coverage
BOC: Breadth of coverage
GATK: Genome analysis toolkit
SNP: Single nucleotide polymorphism
INDEL: Insertion-deletion variation
EDTA: Ethylenediaminetetraacetic acid
BQSR: Base quality score recalibration
VQSR: Variant quality score recalibration
