# Supplementary material for: A comparison of BeadChip and WGS genotyping outputs using partial validation by sanger sequencing
Source: BMC Genomics. 2020 Sep 10;21(Suppl 7):528. doi: 10.1186/s12864-020-06919-x (PMC7488117; doi:10.1186/s12864-020-06919-x)
Supplement: Supplementary file 1 — Additional file 1. FastQC report for forward reads. [file 12864_2020_6919_MOESM1_ESM.html]

sample\_001\_R1.fastq.gz FastQC Report 

FastQC Report

Чт 5 июл 2018  
sample\_001\_R1.fastq.gz

## Summary

- Basic Statistics
- Per base sequence quality
- Per tile sequence quality
- Per sequence quality scores
- Per base sequence content
- Per sequence GC content
- Per base N content
- Sequence Length Distribution
- Sequence Duplication Levels
- Overrepresented sequences
- Adapter Content

## Basic Statistics

| Measure | Value |
| --- | --- |
| Filename | sample\_001\_R1.fastq.gz |
| File type | Conventional base calls |
| Encoding | Sanger / Illumina 1.9 |
| Total Sequences | 399036358 |
| Sequences flagged as poor quality | 0 |
| Sequence length | 150 |
| %GC | 41 |

## Per base sequence quality

## Per tile sequence quality

## Per sequence quality scores

## Per base sequence content

## Per sequence GC content

## Per base N content

## Sequence Length Distribution

## Sequence Duplication Levels

## Overrepresented sequences

| Sequence | Count | Percentage | Possible Source |
| --- | --- | --- | --- |
| ATCGGAAGAGCACACGTCTGAACTCCAGTCACCGCTCATTATCTCGTATG | 661378 | 0.16574379420333424 | TruSeq Adapter, Index 2 (97% over 36bp) |

## Adapter Content

Produced by FastQC (version 0.11.7)
